# A model-informed method to retrieve intrinsic from apparent cooperativity and project cellular target occupancy for ternary complex-forming compounds†

**DOI:** 10.1039/d2cb00216g

**Published:** 2023-05-19

**Authors:** Richard R. Stein, Marianne Fouché, Jeffrey D. Kearns, Hans-Joerg Roth

**Affiliations:** a Novartis Institutes for BioMedical Research Basel Switzerland richard.stein@novartis.com hans-joerg.roth@novartis.com; b Novartis Institutes for BioMedical Research Cambridge MA USA

## Abstract

There is an increasing interest to develop therapeutics that modulate challenging or undruggable target proteins *via* a mechanism that involves ternary complexes. In general, such compounds can be characterized by their direct affinities to a chaperone and a target protein and by their degree of cooperativity in the formation of the ternary complex. As a trend, smaller compounds have a greater dependency on intrinsic cooperativity to their thermodynamic stability relative to direct target (or chaperone) binding. This highlights the need to consider intrinsic cooperativity of ternary complex-forming compounds early in lead optimization, especially as they provide more control over target selectivity (especially for isoforms) and more insight into the relationship between target occupancy and target response *via* estimation of ternary complex concentrations. This motivates the need to quantify the natural constant of intrinsic cooperativity (*α*) which is generally defined as the gain (or loss) in affinity of a compound to its target in pre-bound *vs.* unbound state. Intrinsic cooperativities can be retrieved *via* a mathematical binding model from EC_50_ shifts of binary binding curves of the ternary complex-forming compound with either a target or chaperone relative to the same experiment but in the presence of the counter protein. In this manuscript, we present a mathematical modeling methodology that estimates the intrinsic cooperativity value from experimentally observed apparent cooperativities. This method requires only the two binary binding affinities and the protein concentrations of target and chaperone and is therefore suitable for use in early discovery therapeutic programs. This approach is then extended from biochemical assays to cellular assays (*i.e.*, from a closed system to an open system) by accounting for differences in total ligand *vs.* free ligand concentrations in the calculations of ternary complex concentrations. Finally, this model is used to translate biochemical potency of ternary complex-forming compounds into expected cellular target occupancy, which could ultimately serve as a way for validation or de-validation of hypothesized biological mechanisms of action.

## Abstract 2

Estimating intrinsic from apparent cooperativities to calculate concentrations of ternary complexes in biochemical and cellular assays.

## Graphical abstract

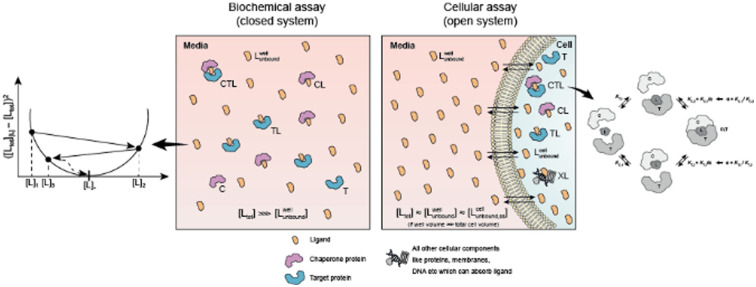

## Introduction

Addressing disease relevant but challenging targets by recruiting them into ternary complexes has become very popular over the last two decades.^1–4^ Collectively, all these efforts are applying various mechanistic concepts to investigate how targets can be modulated by bringing them into proximity to other proteins or by stabilizing the interaction with their native partners. Specifically, targets can be inactivated by drawing them into a *de novo* target–chaperone complex, which disrupts their native protein–protein interaction (PPI).^5–12^ By contrast, targets can be stabilized in their native interactions by molecular glues with the corresponding consequences on their biological function (*e.g.* stimulation).^7,13–19^ By recruitment of target proteins into ternary complexes with ligases, their own ubiquitination and subsequent degradation can be induced^20–30^ or, conversely, they can be rescued from degradation by forming ternary complexes with deubiquitinating enzymes.^31^ Other novel concepts are lysosome-targeting chimeras (LYTACs),^32^ chaperone-mediated autophagy (CMA),^33^ autophagy-targeting chimeras (AUTACs),^34^ proximity-induced phosphorylation^35^ and dephosphorylation^36^ or induced self-association.^1,37^ All approaches share the compound-dependent formation of a ternary complex and aim finally at an inhibition or stimulation of target-related biological signals.

A ternary complex-forming compound can be characterized by three features: (1) its affinity to an assisting protein (here denoted with C for chaperone), which can be either a chaperone like FKBP12, CypA, 14-3-3, a ligase such as CRBN, VHL or a de-ubiquitinase, or any protein that modulates the target in a compound-dependent manner without being directly involved in the native target signalling; (2) its direct and independent affinity to the target protein; (3) its ability to induce positive or negative cooperativity (see definition below) to the ternary complex formation.

**Fig. 1:**
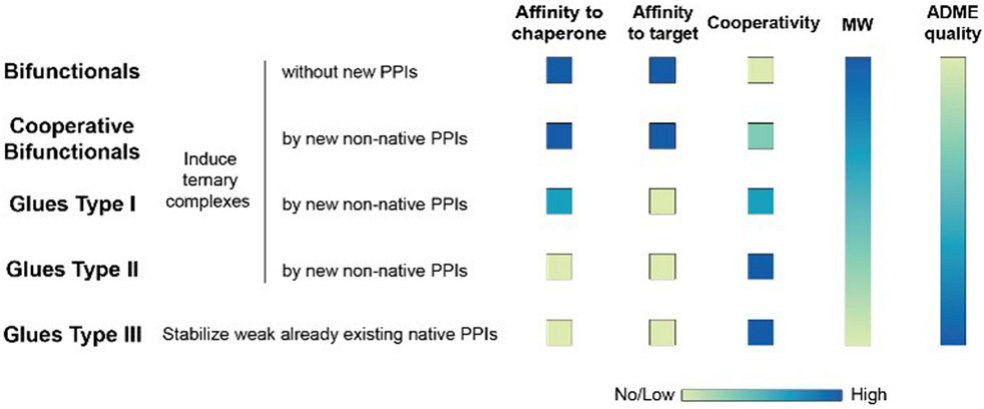
Ternary complex-forming compounds can be grouped into multiple subtypes by their affinities to one or both proteins that constitute the ternary complex and the degree of induced positive or negative cooperativity. The proposed classification is idealized. In practice, ternary complex-forming compounds are often hybrids of two different types. In ternary complexes with similar thermodynamic stability, the molecular weight of the ternary complex-forming compound should inversely correlate with increasing cooperativity due to additional direct protein–protein or glue–protein interactions, which occur exclusively in the ternary complex. The colour gradient bars for “MW size” and “ADME quality” are intended to qualitatively illustrate the generally acknowledged trend that molecular weight and ADME quality are also inversely correlated (*i.e.*, “smaller is better”).

To our knowledge, there are – with the exception of de Vink *et al.*^15^ from 2019 – no published examples of fully described ternary complex-forming compounds with a reported intrinsic cooperativity *α* and *K*_d_s to either both proteins or to one protein along with the *K*_d_ between the two proteins. In most cases, only the affinity of the ternary complex-forming compound to one of the proteins and the corresponding apparent affinity (*i.e.*, the EC_50_ shift) in the presence of the second protein at a constant concentration is reported. However, with only one *K*_d_, it cannot be decided whether the observed EC_50_ shift stems from changes in the cooperativity, the unknown affinity to the second protein or the two proteins to each other (or a combination of all). It was only in 2019 when de Vink *et al.* described for the first time an iterative model-based approach to calculate the intrinsic cooperativity *α* from two independent 2D binding experiments. Since then, no further fully characterized examples have been published. In our view, the characterization of ternary complex-forming compounds by their intrinsic cooperativity *α* has not yet gained the necessary attention of the community, which motivated us to publish this study. The proposed classification characterized by types of intrinsic binding affinities and degrees of cooperativity is therefore a proposal for a systematic general and idealized nomenclature of ternary complex-forming compounds, which must be filled with life through a growing data set of fully characterized ternary complex-forming compounds. It is noteworthy to mention that in practice ternary complex-forming compounds are often hybrids of two different types (Fig. 1).

If a compound binds independently and significantly (*i.e.*, for dissociation constants *K*_d_ < 1 μM) to “chaperone” and target proteins, it is called a bifunctional compound. If a compound binds to only one of the two proteins in an independent and significant manner (typically to the chaperone) while drawing a significant proportion of the thermodynamic stability of the formed ternary complex from the cooperativity of the induced ternary complex, it is called a molecular glue of type I. A compound is classified as a molecular glue of type II if it neither binds to chaperone nor to target alone but still forms a stable ternary complex facilitated by a very high cooperativity. If a compound binds to neither the chaperone nor the target but stabilizes an already existing (weak) interaction between these proteins, it is termed molecular glue of type III. For compounds that bind independently and significantly to both chaperone and target and exhibit a significant (*α* > 10) degree of positive cooperativity, the term cooperative bifunctional is used. It is noted that bifunctionals or molecular glues of different subtype, which induce or stabilize ternary complexes with similar thermodynamic stability, tend to have lower molecular weights the higher their cooperativity. Consequently, the ADME quality – as a trend – may increase with increasing cooperativity, which makes a high cooperativity a beneficial feature for ternary complex-forming compounds.

The work presented here has a basis in the foundational papers of Han,^38^ Douglass *et al.*^39^ and de Vink *et al.*^15^ Since we wish to increase the understanding (and ultimately the dissemination) of the concept of cooperativity in the field of ternary complex-related drug discovery, we repeat the mathematical description of cooperative ternary complexes formed by two possible equilibrium pathways, although this has been described already by Han and others. The three former contributions differ from each other in important aspects. Douglass *et al.* describe non-cooperative ternary complex formation by analytical expressions, while de Vink *et al.* use two 2D binding experiments combined with an iterative approach. In contrast, Han uses an iterative approach to determine free ligand concentrations [L] from concentration of total ligand [L_tot_] and derives from there analytical expressions for the ternary complex and all other equilibrium species. All three approaches share the input requirement of two measured binding affinities (as single *K*_d_s or 2D sets of EC_50_s), which can be either the binary affinity of the ternary complex-forming compound to the two proteins or to one protein and the affinity of the two proteins to each other.

Our presented workflow combines elements from the work of de Vink *et al.*^15^ (iteration to assess intrinsic cooperativity *α*) and Han (iterative determination of free ligand and analytical expressions for equilibrium species) and describes how to iteratively assess the intrinsic cooperativity *α* from a single EC_50_ shift if two *K*_d_s of binary binding events are available. Even if one of the two input parameters cannot be measured, which is likely the case for very weak interactions with *K*_d_s > 250 μM, the model can still be used to study the potential impact on concentrations of ternary complexes and all other equilibrium species by assuming exceedingly large *K*_d_s.

While the proposed modeling framework enables the calculation of ternary complexes and all other relevant equilibrium species through all three possible combinations of two considered pathways (through TL and CL or through TL and TC or through CL and TC), we focus our discussion on the modeling of the pathway pattern with input from two binary *K*_d_s to the two proteins (*i.e.*, bifunctional or cooperative bifunctional compounds and molecular glues of type I with only very weak affinity to the target). Molecular glues of type II present a particular challenge as the formation of ternary complexes relies on cooperativity only due to the lack of any significant binary interaction to either of the two proteins (*e.g.*, *K*_C,1_ and *K*_T,1_ > 250 μM) or any intrinsic interaction of the two proteins to each other, but can still be modeled by applying two estimated high *K*_d_s. Further, this is of practical value to the discovery chemist to inform the appropriate concentrations of compounds to use in a screening experiment, ensuring that anticipated high cooperativities can be detected. A future expansion of the proposed model to molecular glues of type III is envisaged by adding the measured intrinsic protein–protein affinity to then include three binary *K*_d_s (formation of initial chaperone–ligand CL, target–ligand TL and chaperone–target CT binary complexes) and all three corresponding ternary complex formation pathways.

**Fig. 2:**
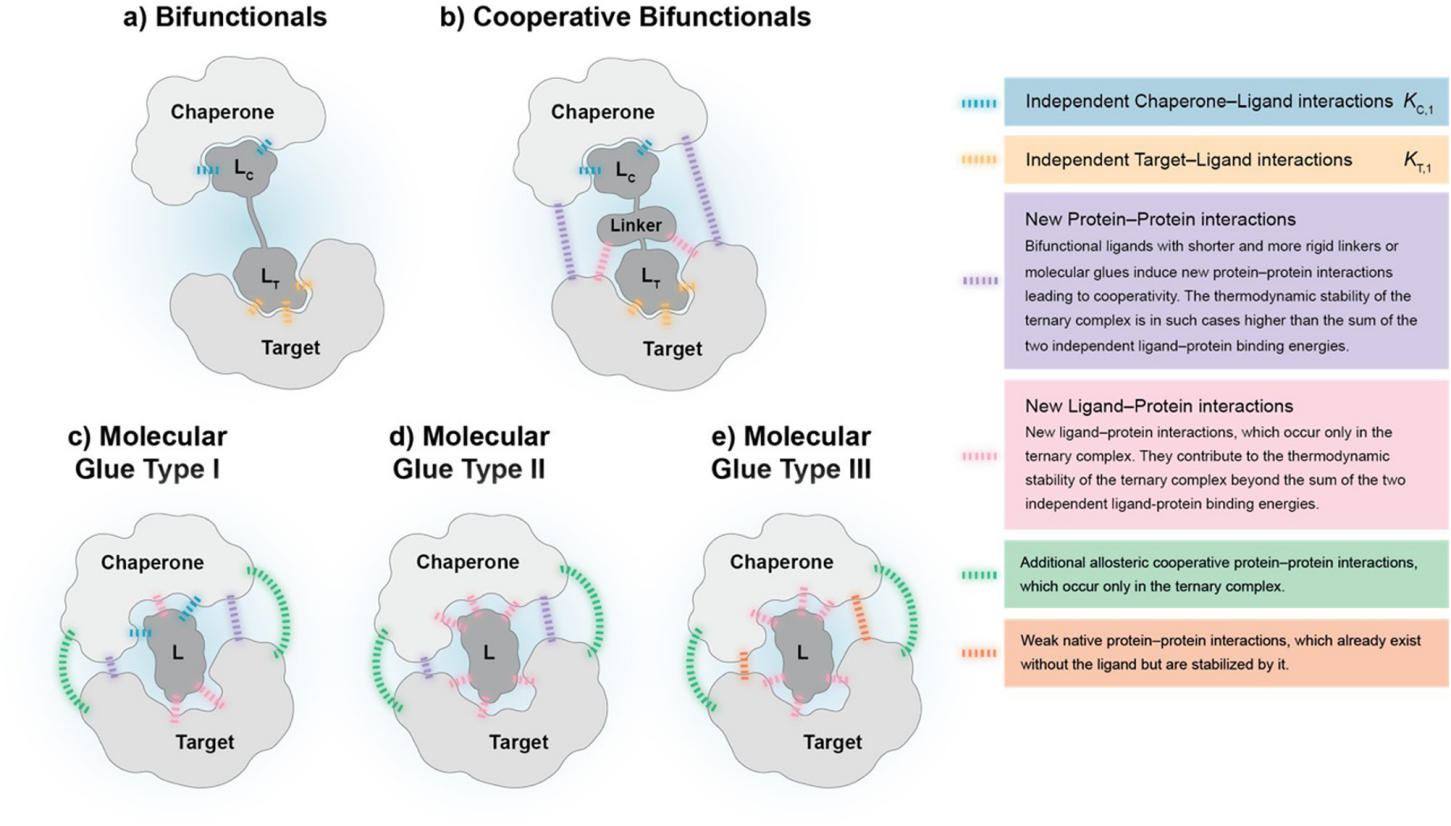
The proposed subtypes of ternary complex-forming compounds can be characterized by their main interactions and the corresponding degree of cooperativity: (a) bifunctionals are molecules with two binding functions of which each binds independently to either a chaperone or a target protein. Ternary complexes that bifunctionals form show no cooperativity (*α* = 1) as no direct target–chaperone, ligand–chaperone or ligand–target interactions are induced *de novo*. (b) Cooperative bifunctionals operate in a similar way as bifunctionals except that they exhibit cooperativity (*α* > 1). They induce direct target–chaperone or ligand–chaperone or ligand–target interactions potentially through more elaborate linkers (shorter, more rigid). The thermodynamic stability of ternary complexes formed by a cooperative bifunctional is higher than the stability resulting from the combination of individual compound affinities to chaperone and target. (c) Molecular glues type I only maintain a measurable intrinsic affinity to the chaperone but not to the target protein. However, ternary complexes from molecular glues type I often induce additional allosteric target chaperone interactions, *i.e.*, protein–protein interactions in distance to the location of recruitment. The thermodynamic stability of the formed ternary complex mostly stems from a significant degree of cooperativity (*α* > 100). (d) Molecular glues type II hold neither affinities to the chaperone nor to the target protein and the two proteins show no intrinsic affinity to each other. The thermodynamic stability of the resulting ternary complex originates from interactions that occur exclusively in the ternary complex, *i.e.*, due to its cooperativity, which is supposed to be high (*α* > 1000). (e) Molecular glues type III are, in contrast to the other subtypes, stabilizing an already existing, typically weak native intrinsic interaction between chaperone and target protein, which adds to the stability of the ternary complex. This type of glues has ideally no measurable intrinsic affinity to neither the chaperone nor the target protein. The mathematical approach to modelling ternary complexes formed by molecular glues of type III and hybrid forms between type I, II and III differ from the here presented and will be discussed elsewhere.

Cooperativity is a continuous value that can vary from negative to positive. From a structural perspective, positive cooperativity can be understood as the contribution to the thermodynamic stability of ternary complexes stemming from newly induced protein–protein and/or compound–protein interactions that exclusively occur when the ternary complex is formed (Fig. 2). Specifically, the increase in cooperativity represents the incremental gain in thermodynamic stability as characterized by the Gibbs free energy of the ternary complex, Δ*G* = *RT* ln *K*_CLT_, relative to the sum of the individual independent potentials (or the product of affinities) of the ternary complex-forming compound (denoted as “L”) to the chaperone (denoted as “C”) and the target (denoted as “T”), 
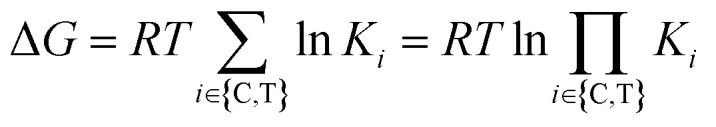
. Since an increase in cooperativity results in a deeper structural integration of the three components L, C and T into the complex, higher selectivity for targets over their similar isoforms can be achieved more easily compared to direct ligand binding. This deeper integration involves additional direct protein–protein interactions (also allosteric ones) across the whole interface of the two proteins, which makes them much more susceptible to small differences in the protein sequence of target isoforms.^20,33,40–43^

Assessing the intrinsic cooperativity *α* in a three-component system has several benefits in a drug discovery program. For a medicinal chemist, it enables the ranking and prioritization of ternary complex-forming compounds that induce desired interactions that only occur in the ternary complex. For a biologist or pharmacologist, it enables the estimation of expected ternary complex concentrations within *in vitro* and cellular systems, and potentially within *in vivo* systems. Herein, we describe a rigorous, computational method that can be applied to both contexts.

## Estimation of ternary complex concentrations *via* a mathematical binding model

**Fig. 3:**
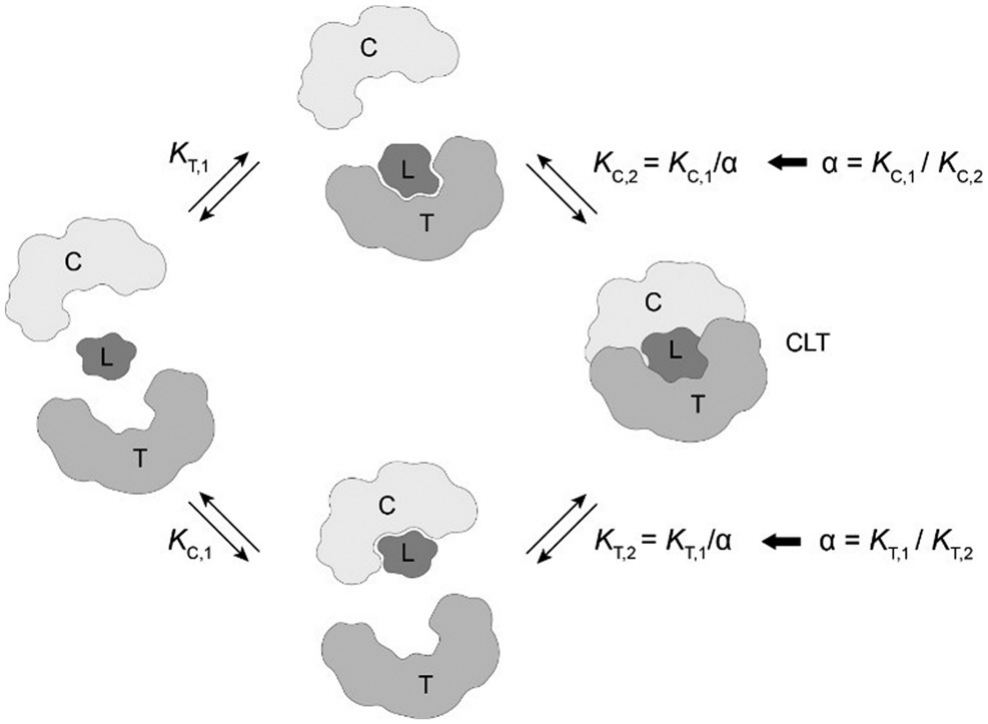
The two main pathways that lead to the formation of the ternary complex CLT are passing through the formation of binary complexes CL and TL. The direct formation of CLT without the intermediate step of forming binary complexes is not considered here. Not discussed is a fourth case, in which the two proteins T and C form at first a binary protein complex TC then followed by a stabilizing ligand binding into CLT. The cooperativity *α* is defined as the ratio of the binary dissociation constants *K*_C,1_ (or *K*_T,1_) of ligand L and C (or T) to *K*_C,2_ (or *K*_T,2_) when ligand L is already prebound to T (as TL) or C (as CL).

The computational method is based upon an understanding of thermodynamics principles and reaction kinetics. First, we will describe the full computational reaction scheme that considers the formation of binary complexes and the subsequent formation of the ternary complex (Fig. 3). From a thermodynamic perspective, the cooperativity of a closed, three-component system is defined as the ratio of the dissociation constant of the free ternary complex-forming compound L and its chaperone C (*K*_C,1_) and the dissociation constant of L and its chaperone C when L is already bound to the target in the binary complex TL (*K*_C,2_).^15,38,39^ Due to the path independence of Gibbs free energy, the analogue ratio of *K*_T,1_ and *K*_T,2_ must yield the same value which is then defined as the intrinsic cooperativity *α*. To mathematically formalize this statement, the formation of ternary complex CLT is denoted at first *via* formation of the binary TL complex (with *K*_T,1_ = [L][T]/[TL] followed by *K*_C,2_ = [TL][C]/[CLT]):
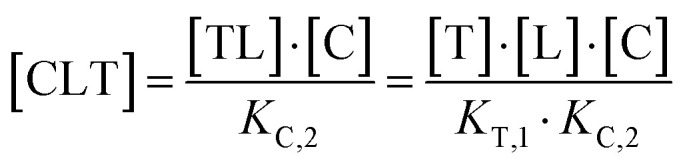
and subsequently, through the parallel pathway, *via* formation of the binary CL complex (with *K*_C,1_ = [L][C]/[CL] followed by *K*_T,2_ = [CL][T]/[CLT]):
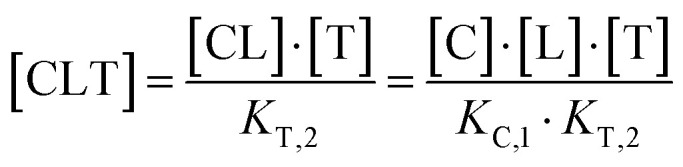
Due to pathway independence, both concentrations of CLT are supposed to be equal, and division of the right-hand side identities then leads to the definition of the intrinsic cooperativity factor *α*:1
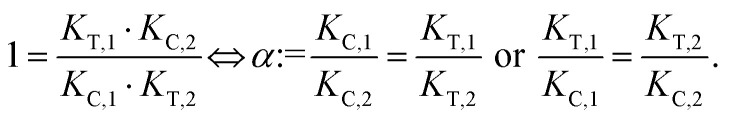
Consequently, for a system with given ternary complex concentration [CLT], an increase in *K*_C,1_ needs to be compensated by lowering *K*_T,2_ and *vice versa*, ergo, the ratio of the binary dissociation constants *K*_T,1_ and *K*_C,1_ and of *K*_T,2_ and *K*_C,2_ are inversely proportional. This method assumes negligible spontaneous ternary complex formation as a simultaneous three-body event without involving the binary complexes of chaperone–ligand CL or target–ligand TL.

Since the intrinsic cooperativity *α* is defined as the ratio of the dissociation constants ([*K*_C,1_ to *K*_C,2_] or [*K*_T,1_ to *K*_T,2_]), a positive cooperativity value *α* > 1 indicates that the binding of free ligand to target is weaker by itself than when ligand is bound by chaperone (the binary complex CL). By symmetry, the same also applies to the other path to ternary complex formation: if *α* > 1 then *K*_C,1_ > *K*_C,2_, meaning that binding of free L to C is weaker by itself than when L is already prebound to T as TL. Based on these considerations, three extreme scenarios are contemplated. First, if a compound has no measurable affinity to target (*i.e.*, *K*_T,1_ is above a detectable limit) but still induces a ternary complex of a given thermodynamic stability, *K*_C,2_ is assumed to be very low. However, as *K*_C,2_ can only be very low if *α* is very high, this means that the formation of the binary complex with chaperone (governed by *K*_C,1_ and *α*) dominates the formation of the ternary complex. In this case, the only significant pathway to the ternary complex is *via* the chaperone–ligand complex CL. Second, in the scenario of non-cooperative binding (*α* = 1), the affinity of the free (unbound) ligand to chaperone remains the same irrespective of whether the ligand is prebound to target or not (and *vice versa*). And third, it is noted that if there is no cooperativity (*α* = 1) and no measurable affinity to the second protein, then only the binary complexes (CL or TL) will form.

Next, we will describe how the proposed method accounts for the inability to experimentally measure all parameters in the full binding model. According to the above relationships, determining the system's cooperativity *α* would require measurement of the formation of the ternary complex from the intermediary, binary complexes to characterize the *K*_C,2_ or *K*_T,2_ parameters. While measurement of the *K*_C,1_ or *K*_T,1_ parameter is achievable with routine biochemical and biophysical assays, direct measurement of *K*_C,2_ or *K*_T,2_ is challenging due to the confounding factor of the equilibrium of the binary complexes (CL or TL). The estimation of *K*_C,2_ or *K*_T,2_ would therefore require calibrated analytical signals for all three equilibrium components for each ternary complex-forming path (CL, free T, and CLT; TL, free C, and CLT).

However, the fact that the intrinsic cooperativity *α* is identical along both paths allows the elimination of *K*_C,2_ or *K*_T,2_ by terms consisting only of the binary (*K*_C,1_ or *K*_T,1_) and the cooperativity *α*:2
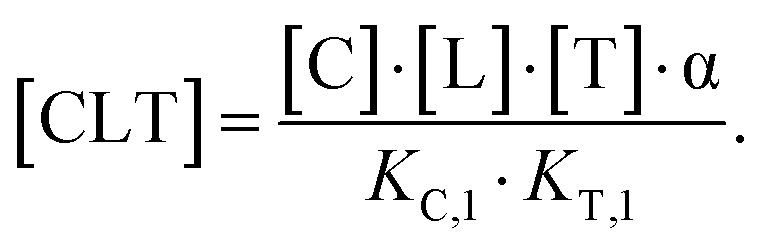
This equation defines the concentration of ternary complex CLT as a function of the measurable binary affinities (*K*_C,1_ and *K*_T,1_), the cooperativity *α*, and the concentrations of the free components (ligand, chaperone, and target). As concentrations of the free components are typically inaccessible in biochemical assays, except at the inflection point of a binary binding experiment in which [C] ≡ [CL] ≡ [C_tot_]/2 or [T] ≡ [TL] ≡ [T_tot_]/2, we instead substitute the corresponding total concentrations [L_tot_], [C_tot_], and [T_tot_]. In a previous study, Douglass *et al.*^39^ have shown that in cooperative systems (*α* ≠ 1) algebraic expressions of the ternary complex concentration [CLT] in terms of cooperativity *α*, affinities, and total concentrations [L_tot_], [C_tot_] and [T_tot_] cannot be obtained. However, numerical solutions are found by reducing (eqn (2)) to contain only the four measurable and monitorable variables and estimate the otherwise inaccessible free ligand concentration [L] from [L_tot_] by adding an additional non-linear equation^44–46^

**Fig. 4:**
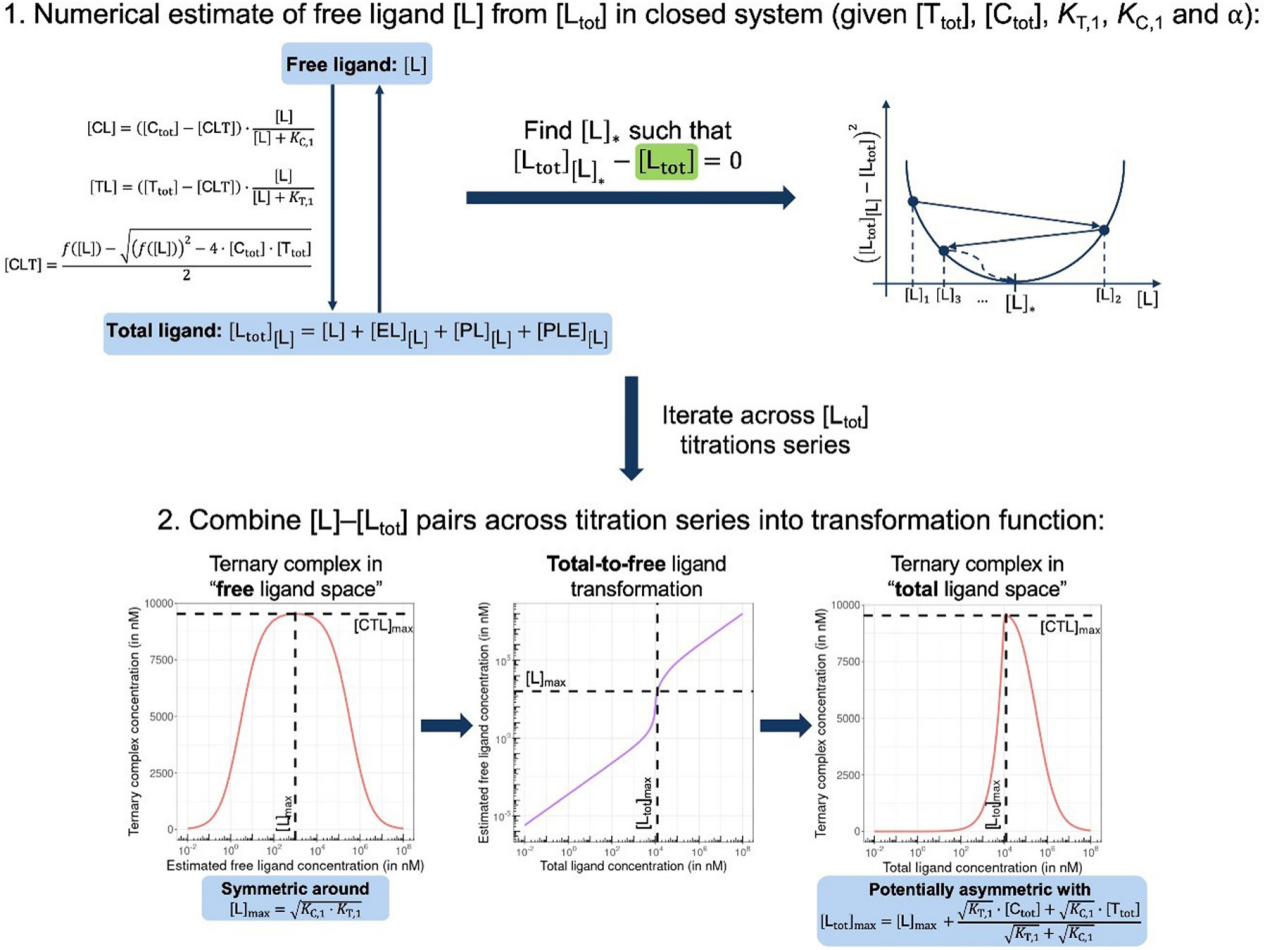
In closed systems like biochemical assays, the otherwise hard to determine free ligand concentration [L] is iteratively estimated by matching the given [L_tot_] with [L_tot_]_[L]_ = [L] + [CL]_[L]_ + [TL]_[L]_ + [CLT]_[L]_ that involves all individual species equations for CL, TL and CLT (top). When the ternary complex concentration is drawn with respect to free ligand concentrations, this curve is symmetric around the maximizing free ligand concentration [L]_max_ (bottom left). Depending on the chosen parameters, the corresponding total-to-free ligand transformation can show a high degree of non-linearity (bottom middle) resulting in a potential asymmetry of the ternary complex concentration curve with respect to total ligand (bottom right). Moreover, the total ligand concentration referring to the maximum of ternary complex [L_tot_]_max_ is predicted to deviate from [L]_max_. Compound parameters are referring to a standard cooperative bifunctional with *K*_T,1_ = 10 000 nM, *K*_C,1_ = 100 nM and *α* = 16; environmental parameters of [C_tot_] = 25 000 nM and [T_tot_] = 10 000 nM are representative of a biophysical assay.

Accordingly, to reduce (2), the identity [C_tot_] = [C] + [CL] + [CLT] is used with 
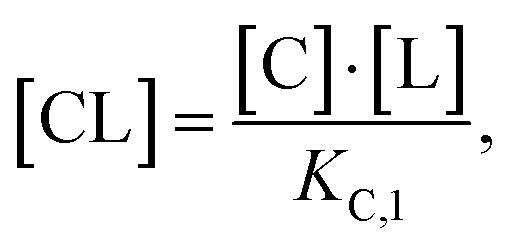
 which yields:
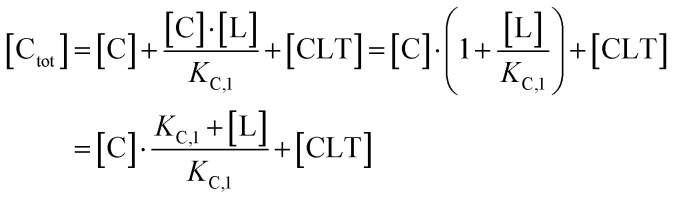
with expressions for the free chaperone and the chaperone–ligand complex:3
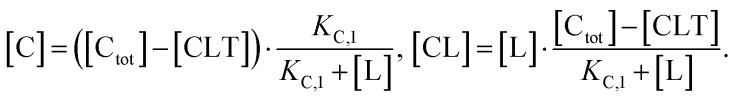
The analogous equation for the free target and the target–ligand complex is:4
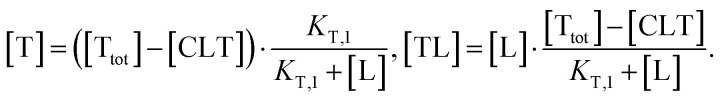
Substituting both free chaperone and target into (2) yields:5
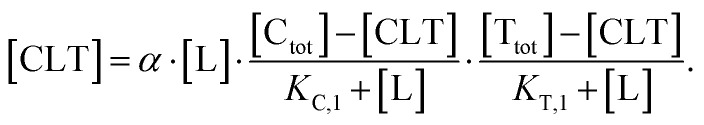
From this, a quadratic equation for [CLT] in dependence of only known [C_tot_] and [T_tot_] concentrations, measurable *K*_C,1_ and *K*_T,1_ and intrinsic cooperativity *α* is found with only one meaningful solution:^38^6
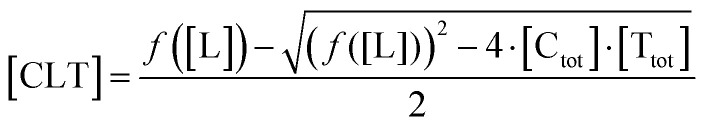
for 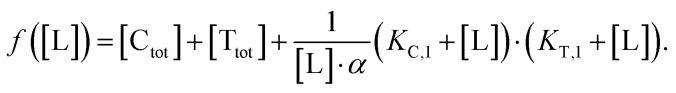 In order to estimate free ligand concentrations from given [L_tot_] an additional non-linear equation needs to be solved:Find [L] > 0 such that [L_tot_]_[L]_ − [L_tot_] = 0 while [L_tot_]_[L]_ = [L] + [CL]_[L]_ + [TL]_[L]_ + [CLT]_[L]_,which is routinely performed using non-linear root finding algorithms. Here [CL]_[L]_ and [TL]_[L]_ denote the free ligand-dependent binary species concentrations from (eqn (3) and (4)). The model-based estimate of the free ligand [L] is then found as the match of free ligand-dependent [L_tot_]_[L]_ (for given [C_tot_], [T_tot_], *K*_C,1_, *K*_T,1_, *α*) and the given total ligand [L_tot_] (Fig. 4, panel 1). An alternative to our approach, which is adapted from Han,^38^ has been recently proposed^15^ and involves solving one nonlinear equation per species, potentially offering more flexibility to include additional constraints, such as tracer equilibria.

From (5), the shape of the [CLT] curve as a function of free ligand [L] can be deduced (Fig. 4, panel 2). The [CLT] curve is symmetric around a maximum free ligand concentration of 
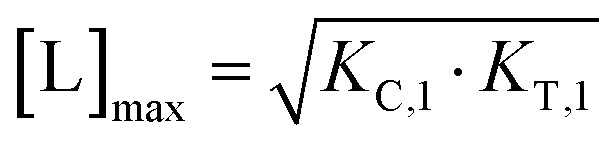
 and approaches 0 for free ligand concentrations approaching 0 or infinity. This resulting symmetric bell-shaped curve of ternary complex concentrations as a function of free ligand is also referred to as “hook effect”. However, distortions from this symmetry are observed when changing into the [L_tot_] reference system (*e.g.*, when [CLT] is drawn against [L_tot_] instead of free ligand [L]).^39^

The intrinsic cooperativity represents a natural constant that characterizes the degree of structural integration of the three components C, L and T into a ternary complex CLT by a simple unit-less number. This is therefore a functional attribute that can be subject to optimization during compound lead optimization. Ternary complex-forming compounds can be optimized in several ways. First, optimization towards a higher potency (*i.e.*, a lower potential *K*_CLT_ = *K*_C,1_·*K*_T,2_) may ultimately result in a lower minimal efficacious dose prediction. Second, optimization towards higher cooperativity relative to direct target binding (*e.g.*, from bi-functional towards more “glue-like” properties) may enable the use of a lower molecular weight compound and may also allow exploration of improved ADME and target selectivity properties (Fig. 1). In these ways, explicit determination of the value of the intrinsic cooperativity enables the direct ranking of ternary complex-forming compounds irrespective of the assays used. Notably, this provides broader utility than relative measures like *A*_max_ with their dependency on experimental conditions. In analogy to the potency optimization of binary ligands, where a rigidification strategy, which reduces entropy costs, is typically preferable over aiming for additional interactions due to an anticipated gain in molecular weight, increases in cooperativity are considered preferable for ternary complexes over those in direct target binding (*i.e.*, *K*_T,1_; Fig. 3) – although both can contribute to an overall higher stability of the ternary complex. As the derivatization strategy for optimizing either of the two parameters is different, it is crucial to understand their impact on a quantitative basis. Knowledge of the quantitative contribution of the cooperativity *α* to the formation of a ternary complex (*vs.* the contribution of direct binding) provides a step towards a more complete perspective on hits from molecular glue screenings. Although these more “glue-like” hits may show at first weaker thermodynamic stability in the ternary complex relative to bi-functional ternary complex-forming compounds – as suggested by lower *A*_max_ values, which do not allow to distinguish whether a gain in potency is due to an increase in cooperativity or direct target binding – they do have potential for higher intrinsic cooperativity *α*. Such hits may be considered more promising for further optimization, particularly if a high selectivity over target isoforms is envisaged. This highlights the need for a method to retrieve the cooperativity *α* of a ternary complex from the two easily and offline measurable binary affinities, which is described below.

## Why and how to translate potency from biochemical to cellular assays for ternary complexes

In drug discovery, the translation of measured biophysical or biochemical potency to cellular target engagement is a long-standing but unsolved problem for binary complexes and is further confounded for ternary complexes. This stems from the fact that protein concentrations in biophysical and cellular assays are often, for practical reasons, selected to be very different. In particular, biophysical assays monitoring ternary complexes often operate with a large excess of target over chaperone protein and/or with protein concentrations above *K*_d_s. This problem is even more pronounced for ternary complexes when the combinatorial issues of intermediary binary complexes are fully considered. For example, for ternary complexes, the concentrations and the ratio of the two involved proteins and their affinities significantly influence the overall relationship of *K*_CLT_ = *K*_C,1_·*K*_T,2_. In this section, we describe a novel method to predict cellular potency from biochemical assay data.

Translation of biophysical or biochemical potency to cellular activity can indicate when a tested compound is sufficiently optimized to elicit the maximal desired level of cellular response. This is most easily measured at ligand concentrations at which 100% of cellular target protein is bound into the ternary complex and where cellular activity may be reasonably expected to also be saturating. If no or insufficient cellular activity is observed under these saturating conditions, then this may highlight a need for the project team to revisit the underlying mechanistic hypothesis. However, it may not be biologically possible to achieve 100% target occupancy within a cell, in which case it will also be important for the drug discovery team to characterize the precise relationship between target occupancy and cellular response to inform drug candidate selection and, eventually, the anticipated human dose. While not explicitly described in this manuscript, our proposed method can provide valuable insights *via* the model-based prediction of ternary complex concentrations across a range of compound (ligand) concentrations and population variability in target and chaperone levels. However, the proposed method to estimate intracellular equilibrium concentrations of ternary complexes assumes measurable cellular concentrations of C and T being the monomeric species, which might not always be fully true.

**Fig. 5:**
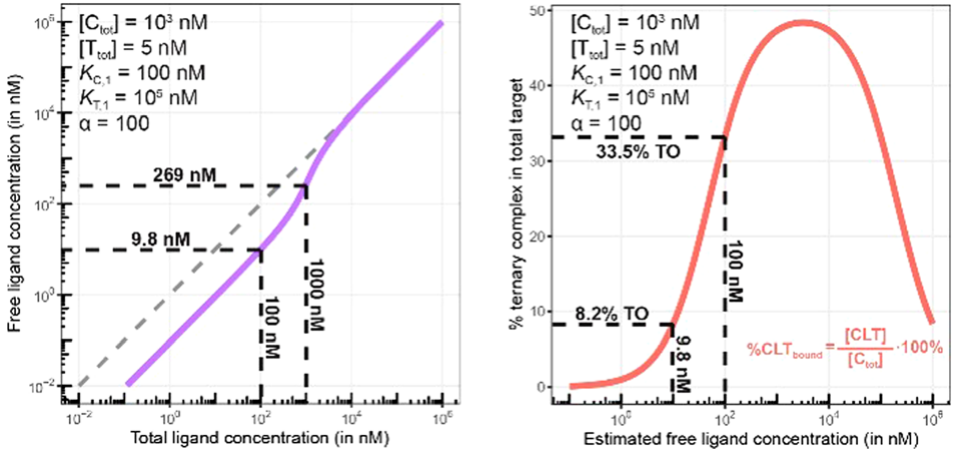
Left: A model-based correlation between concentrations of total and free ligand [L_tot_] and [L] = [L^well^_unbound_] (purple line) shows strong deviation from the identity line (stippled line). Simulations were performed with the indicated values of experimental parameters [C_tot_], [T_tot_], *K*_C,1_, *K*_T,1_ and cooperativity *α* representative of a biochemical assay. The two values indicated on the plot (9.8 nM and 269 nM) are the calculated [L^well^_unbound_] when 100 or 1000 nM [L_tot_] is applied, respectively. Right: The comparison of the same total ligand concentration [L_tot_] in a biochemical and a cellular assay to assess the influence of the type of assay on the expected target occupancy. The two values indicated on the plot (8.2% and 33.5%) are the expected steady state target occupancy in a cellular assay when a [L_tot_] = [L^well^_unbound_] = 9.8 or 100 nM is applied to the well, respectively.

Biophysical assays for characterizing ternary complex-binding constitute a closed system with three initial species of ligand, chaperone and target that are homogeneously dissolved in a single compartment. To apply the model and predict ternary complex concentrations, the required inputs are the cooperativity *α*, the binary dissociation constants *K*_C,1_ and *K*_T,1_, the total concentrations of chaperone and target ([C_tot_] and [T_tot_]), and the concentration of free ligand [L]. For illustrative purposes, characteristic parameters are: [C_tot_] = 1000 nM, [T_tot_] = 5 nM, *K*_C,1_ = 100 nM, *K*_T,1_ = 100 000 nM, and cooperativity *α* = 100. The free ligand concentrations [L] that are computed by the above presented iterative procedure deviate significantly from the total ligand concentration [L_tot_] emphasizing the importance of understanding the relationship of free and total ligand, (Fig. 5, left). As two examples using these parameters, an [L_tot_] = 1000 nM converts into [L] = 269 nM, while an [L_tot_] = 100 nM is estimated to yield a nonlinear decrease to less than 10 nM free ligand concentration.

Cellular assays differ from biophysical assays not only in terms of the concentrations for target and chaperone but most importantly a cellular assay is an open system consisting of a well compartment (*i.e.*, extracellular media) containing only drug and medium which is in exchange with the cells. The influx of unbound compound from the well into the cell results in a dynamic equilibrium between the extracellular medium and the intracellular compartment, with the assumption that the cellular uptake reaches steady state (ss) within the assay incubation time. Assuming that the extracellular media volume is much greater than the total intracellular volume (typically by factor 1000), free intracellular ligand concentrations in steady state roughly correspond to the incubated total and the free/unbound ligand concentrations in the well^44–46^[L^well^_tot_] ≈ [L^well^_unbound_] ≈ [L^cell^_unbound,ss_].Under this formalism, all cellular binding events beyond the intended binding to the target do not affect [L^cell^_unbound,ss_] at equilibrium and can hence be ignored. The underlying theory for this critical simplification is described in detail, below.

First, a relationship between extracellular and intracellular ligand levels must be established. Assessing the intracellular free ligand concentration [L^cell^_unbound,ss_] by means of a model is challenging as cellular uptake rates, unspecific membrane and intracellular protein binding and efflux rates are additional factors beyond the binary and ternary complex-forming equilibria with the chaperone and target protein. However, the quantification of all these numerous interfering events is not necessary when the boundary condition of a large excess of unbound ligand in the well is considered. While the concentration of intracellular free ligand [L] (=[L^cell^_unbound,ss_]) is the result of unknown simultaneous binding events (all with unknown dissociation constants), this value simplifies to [L^well^_unbound_] for well volumes that are exceedingly larger than the total cellular volume (*e.g.*, in the range of factor 1000) and consequently the media compartment acts as an “infinite” reservoir.

Second, this relationship must be extended to the equivalency of the unbound ligand concentrations. For a ligand that is sufficiently chemically and metabolically stable within the incubation time of the assay, [L^cell^_unbound_] and [L^cell^_bound_] are maintained at constant levels when the cellular uptake has reached steady state. To experimentally verify that the steady state in the cellular uptake assay has been reached, only [L^cell^_tot_] must be monitored over time until it plateaus^45,47,48^ In this case, a differentiation between [L^cell^_unbound_] and [L^cell^_bound_] is not required because all occurring binding events are in equilibrium and, thus, [L^well^_unbound_] corresponds to [L^cell^_unbound_].

**Fig. 6:**
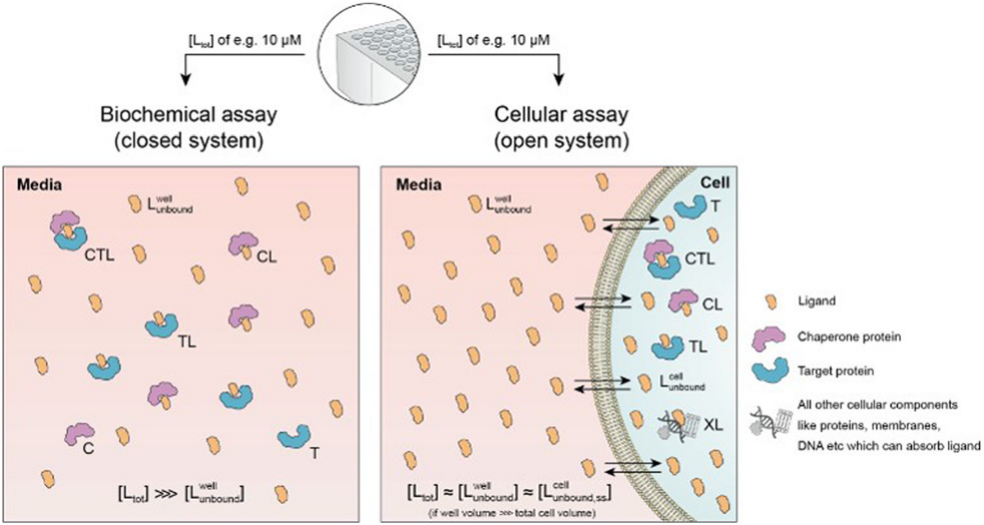
Biochemical and cellular assays differ in various aspects – most importantly, the biochemical assay represents a closed system whereas the cellular assay is considered an open system. In a biochemical assay that monitors ternary complex formation, the three species of chaperone C, target T and ligand L are homogenously dissolved and in equilibrium with each other. As ligand is incorporated into the binary (CL and TL) and ternary (CLT) complexes, the free ligand concentration in the well [L^well^_unbound_] can become significantly lower than the initially applied [L_tot_], depending upon the assay conditions. In contrast, a cellular assay is an open system due to the exchange between the cell and the media in the well. As free ligand can permeate through the membrane into the cell, [L^cell^_unbound_] increases until it corresponds to [L^well^_unbound_]. For well volumes that are exceedingly larger than the total cell volume, the monitorable [L_tot_] corresponds under equilibrium conditions to [L^well^_unbound_] and the latter to [L^cell^_unbound_].

Third, the steady state free ligand concentrations must be considered. The previous assumption that steady state is reached when [L^cell^_tot_] saturates is even true when the freely exchangeable ligand accumulates in a specific cellular compartment irreversibly as [L^cell^_unbound_] would not be affected. Obviously, when steady state is not yet reached, it is not possible to estimate the intracellular concentration with the presented methodology. This steady state criterion is valid for cellular uptake values ranging from low (MDCK *P*_app_ values <1·10^−6^ cm s^−1^) to high (MDCK *P*_app_ values >5·10^−6^ cm s^−1^) and in the case of metabolic instabilities provided that they do not impair that steady state is reached within the incubation time of the assay. To translate the computational method from a biochemical assay (closed system) to a cellular assay (open system) becomes, despite its higher complexity, easier as the required free intracellular concentrations [L^cell^_unbound,ss_] corresponds to the total ligand concentration in the well [L^well^_tot_]. This then allows the direct calculation of the intracellular concentrations of binary and ternary complexes and the underlying component species in the ternary complex-forming equilibrium (given *K*_C,1_, *K*_T,1_, [C_tot_], [T_tot_] and *α*). (Fig. 6).

The computational method can be used to bridge the conditions in biochemical and cellular assays. As one example, the formation of ternary complexes was modeled with the same parameters as used in the case above (Fig. 5 left). A comparison metric was defined as the percentage of total target that is bound within the ternary complex %CLT_bound_ = [CLT]/[T_tot_]·100%. In the biochemical assay, an [L_tot_] = 100 nM yields an [L^well^_unbound_] = 9.8 nM for [C_tot_], [T_tot_], *K*_C,1_, *K*_T,1_ and *α* as above resulting in a target occupancy of 8.2%. If the same [L_tot_] = 100 nM is applied to a cellular assay (using the same protein concentrations as in the biochemical assay), a steady-state intracellular free/unbound ligand concentration [L^cell^_unbound_] = 100 nM is anticipated, which results in 33.5% of target bound in the cell (Fig. 5, right). To achieve the same value in the biochemical assay (*i.e.*, [L] = [L^well^_unbound_] = 100 nM), an [L_tot_] of approximately 800 nM would be needed (data not shown). Interestingly, the target occupancy in the cellular assay could, for the same applied compound concentration, exceed its analogue in the biochemical assay provided that steady state has been reached and no metabolic instabilities occur. As this example highlights, care must be taken if selecting compounds based only on biochemical assay results. Importantly, for the same total ligand concentration [L_tot_], the free ligand concentrations [L] can deviate strongly between a biochemical and cellular assay, which is expected to have a strong effect on the concentration of all equilibrium species (including the ternary complex) and will ultimately influence the ranking of investigated compounds (Fig. 6).

## How to determine intrinsic from apparent cooperativity

The prior section described how differences in free ligand concentrations can arise between biophysical/biochemical and cellular assays for the same total ligand concentrations [L_tot_]. In this section, a combined workflow of specific binding experiments and model-based simulations is presented to determine the value of the intrinsic cooperativity *α*. This will complete the description of the method to determine ternary complex concentrations, as the derivations of the other parameters (*K*_C,1_, *K*_T,1_, [C_tot_] and [T_tot_]) are described above.

As direct quantification of ternary complexes is often not possible, knowledge of the value of the intrinsic cooperativity *α* is required to computationally predict the concentrations and to guide compound optimization towards higher potency, selectivity, and better ADME properties. Since direct assessment of the intrinsic cooperativity *α* is even more challenging than the direct measurement of concentrations of ternary complexes, the term of apparent cooperativity is introduced. The implicit characterization of apparent cooperativity by the increased affinity between two species in the presence of a third one is a generally accepted concept. In particular, the difference in ligand affinity to a single protein (*K*_C,1_ or *K*_T,1_) *vs.* the ligand affinity to the pre-formed binary protein–protein complex CT has been studied extensively, for instance, in molecular glues of type III that stabilize the interaction of the hub protein 14-3-3*σ* and a fragment of the estrogen receptor *α*.^49^ The novelty of our proposed workflow is to assess the intrinsic cooperativity *α* from only one EC_50_ value derived from one protein concentration measurement in a single binary binding experiment. In this mathematical framework, apparent cooperativity represents the shift of the EC_50_ of the biochemical binding experiment C + L → CL (or T + L → TL) executed in the absence and presence of the counter protein T (or C). The monitored binding, here through protein C, results alone only in formation of CL and in the presence of the counter protein in formation of CL and CLT. As only CLT but not CL (as described by eqn (6) and (3)) depends on the cooperativity *α*, the apparent cooperativities can then be directly used to iteratively determine the intrinsic cooperativity *α* for given binary *K*_d_s *K*_C,1_, *K*_T,1_. Of note, while the intrinsic cooperativity represents a natural constant, the apparent cooperativity is dependent on the system concentrations [C_tot_] and [T_tot_] and will therefore vary based upon experimental conditions.

If a ternary complex-forming ligand has measurable affinities to a chaperone protein and a target protein, two independent pathways can occur that go through the intermediary binary complexes (Fig. 3). Since the intrinsic cooperativity *α* is defined as the ratio of the dissociation constant of the free (unbound) ligand to chaperone or target (*K*_C,1_ or *K*_T,1_) over the dissociation constant of the same binding event but with ligand being already pre-bound in a binary complex (*K*_C,2_ or *K*_T,2_; eqn (1) and Fig. 3), simple binary binding experiments can be used to measure apparent cooperativity and the model then applied to calculate the intrinsic cooperativity.

Specifically, this can be achieved by measuring the counter protein dependent EC_50_ shift of a binary binding curve. To optimally calculate the intrinsic cooperativity *α* from apparent cooperativities, it is helpful if the experimentally induced EC_50_ shift of the binary binding event is large. A recommendation is to monitor this binding through the protein with the weaker affinity to the ligand. In the first experiment, the concentration for the weaker binding protein should be set as low as experimentally tolerated such that an EC_50_ value for the TL (or CL) formation can still be properly recorded. In the second experiment, the same binding assay is repeated but in the presence of the counter protein at a high excess over the other protein. The higher the excess of the counter protein, the greater the effect of the intrinsic cooperativity *α* will be on the apparent cooperativity/EC_50_ shift.

**Fig. 7:**
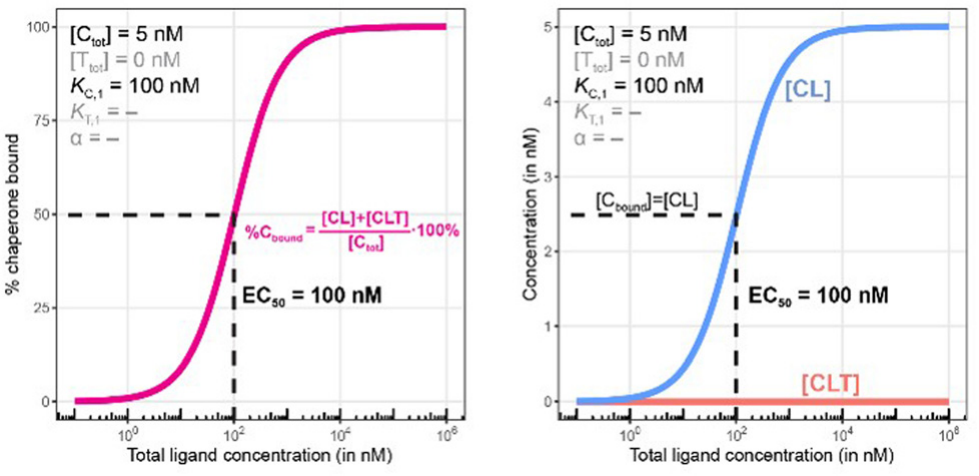
Simulations of bound chaperone without counter protein present ([T_tot_] = 0 nM). Results are shown for percentage of total bound chaperone (left) and for each chaperone-containing complex (CL and CLT, right). As this is a binary binding experiment, total bound chaperone is solely due to formation of the CL complex ([CLT] = 0).

**Fig. 8:**
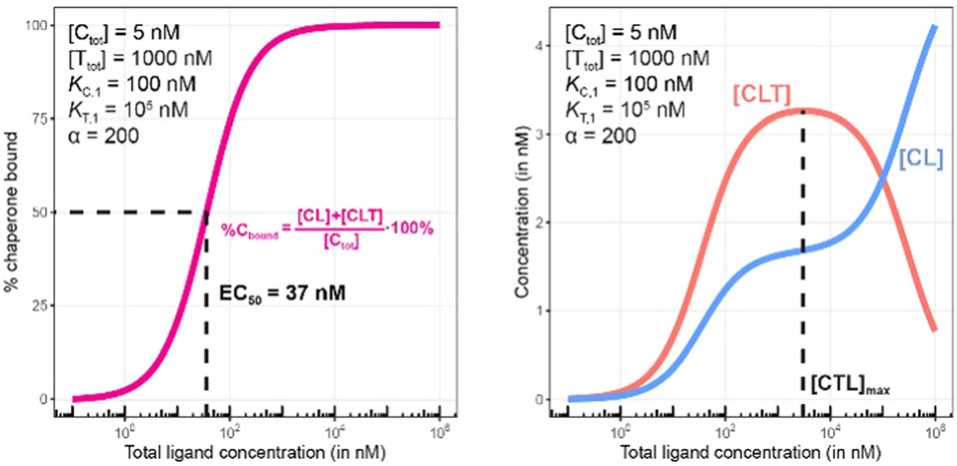
Simulations of bound chaperone, as in Fig. 7, but with counter protein present at a 200-fold excess over chaperone ([T_tot_] = 1000 nM) and a cooperativity of *α* = 2. Results are shown for percentage of total bound chaperone (left) and for each chaperone-containing complex (CLT and CL, right).

Fig. 7 and 8 describe a pair of model simulations to quantify ligand binding under varied counter protein concentrations. As it was described in Fig. 5, the percentage of bound chaperone protein %C_bound_ = [C_bound_]/[C_tot_]·100% for [C_bound_] = [CL] + [CLT] is used as a metric.

In Fig. 7, the model is simulated with no target protein in the reaction ([T_tot_] = 0 nM). In this condition, the binding experiment is a purely binary event (*i.e.*, only C + L → CL), and thus any measured change in the monitored protein C is due to ligand binding, and the observed increase in bound chaperone (left) is the result of CL formation (right). As expected, the %C_bound_ curve coincides with the CL formation curve and no ternary complex CLT is formed.

As this is a system with a fixed total concentration of chaperone [C_tot_], %C_bound_ is proportional to the concentration of bound chaperone [C_bound_] and acts here as its proxy. For this exemplary situation of a binary binding experiment with a low concentration of chaperone ([C_tot_] = 5 nM) and a nominal affinity of ligand to chaperone, the calculated EC_50_ value for %C_bound_ is observed to be equivalent to the *K*_d_ of compound L to C of (*K*_C,1_ = 100 nM) (Fig. 7, left). However, if the protein concentration becomes close to or exceeds *K*_C,1_ this finding will no longer be maintained.

Once the target protein T is added to the system, the model simulations reveal a shift in the binding curve as chaperone C is additionally bound into ternary complex CLT. Now, the two Figure panels (%C_bound_ at left; concentrations of CL and CLT complexes at right) each reveal a different insight (Fig. 7 and 8). The EC_50_ of the %C_bound_ curve, which corresponds to the normalized sum of the CL and CLT formation curve shifts from 100 nM to 37 nM. This shift is facilitated by the higher affinity of present CL to T, relative to the affinity of L to T. The shift of the EC_50_ to the left practically means that there is more bound chaperone %C_bound_ for any given [L_tot_]. The plot of CLT and CL curves reveals an [L_tot_]-dependent competition between the formation of ternary and binary complexes, with a “hook effect” observed in the CLT curve and a “shoulder” binding curve for CL. This is discussed in detail below.

There are several points to be highlighted from the comparison of the simulations with and without the presence of the counter protein: (1) As mentioned above, the addition of target protein T causes a decrease in the EC_50_ of the %C_bound_ curve from 100 nM to 37 nM. This nearly three-fold improvement in bound chaperone demonstrates the impact of positive cooperative binding. (2) Care must be taken in the interpretation of the %C_bound_ curve, as it does not represent CLT nor CL formation alone but rather the sum of all events of L_tot_-dependent binding to the chaperone C (C + L → CL, CL + T → CLT and C + TL → CLT). In other words, the %C_bound_ curve cannot be interpretated as a CLT formation curve. (3) There is an L_tot_-dependent relationship between the formation of the two binary complexes (CL and TL) and of the ternary complex (CLT). This is observed as both a “hook effect” in the CLT curve and a “shoulder” in the CL curve and can best be explained in three phases. At low [L_tot_] concentrations where the chaperone and target proteins are both in excess, the binary and ternary complexes readily form. At intermediate [L_tot_] concentrations, the three components are in balance (relative to the affinity parameters) and the high cooperativity value (*α* = 200) favors the ternary complex. At high [L_tot_], the ligand is in excess over the other components. This saturates the free chaperone and target protein in binary complexes and thereby removes the paths to formation of the ternary complex (hence, the hook effect in the CLT curve). Eventually, CL becomes the only contributor to the %C_bound_ curve. (4) The [L_tot_] that corresponds to the maximum ternary complex concentration [L_tot_]_max_ is an important concentration to measure. This represents a potential optimal concentration for drug activity. Under these conditions, the [L_tot_] value is found to be 5000 nM, where approximately 66% of chaperone C is bound in the ternary complex and 33% in the CL binary complex.

Since in experimental practice all parameters of this system, namely *K*_C,1_, *K*_T,1_, [C_tot_] and [T_tot_], are known, the value of the intrinsic cooperativity *α* is the only unknown parameter to be estimated. It is required that values of both *K*_C,1_ and *K*_T,1_ be known to estimate a unique value for intrinsic cooperativity *α*. If only one value is known, the observed EC_50_ shift can be either due to a change in intrinsic cooperativity, ligand binding, or a combination of both. This concept is known as parameter identifiability and is a standard consideration within the mathematical modeling field. Parameter estimation can be performed in two ways, with the first being heavily favored. When multiple binding curves are measured using a dilution series of different protein concentrations, standard model parameter fitting techniques can be applied. This has the advantage of determining a robust parameter estimation even for observed non-linearities in the binding curve. However, often, only one binding curve is produced under a single set of conditions. In this second case, it is also possible to manually determine the parameter value if a simulation tool is made available to the scientist (see ESI†). A quick assessment can be performed within the tool by entering the values of the known parameters and then repeatedly simulating the model while varying the value of the *α* parameter. By applying this manual workflow to the above example, the intrinsic cooperativity *α* would be increased starting from *α* = 1 until the simulated and experimentally measured %C_bound_ curves are – by incremental shifts to the “left” – matching. At this point, the EC_50_ of the simulated curve should be 37 nM while the *α* value corresponds to 200.

Importantly, the observed EC_50_ shift of the %C_bound_ curve by a factor of 2.7 (from 100 nM to 37 nM) is not the intrinsic cooperativity *α*, which is a natural constant, but its protein concentration-dependent representation. This factor is instead termed apparent cooperativity and is defined as the ratio between the two EC_50_s (100/37 = 2.7).

**Fig. 9:**
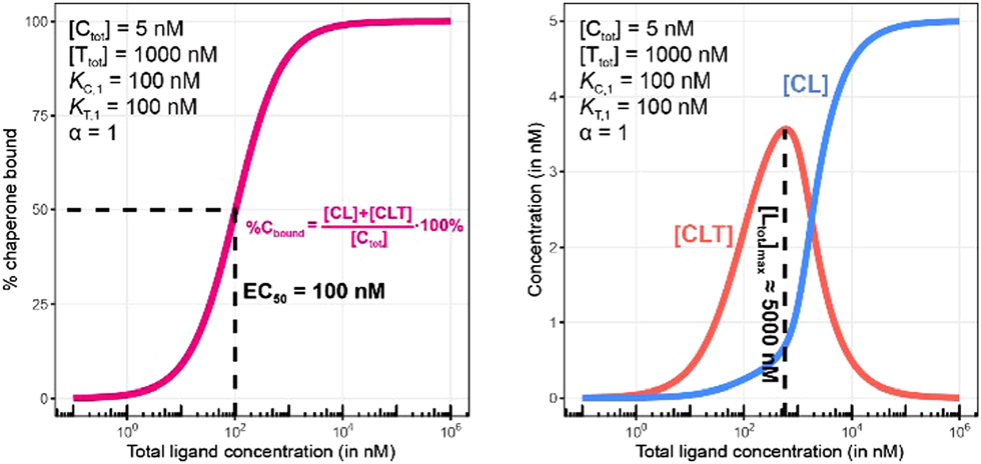
Simulations of bound chaperone, as in Fig. 7, but now configured as a non-cooperative system (*α* = 1) and an equivalent affinity for each binary complex (*K*_C,1_ = *K*_T,1_ = 100 nM). Results are shown for percentage of total bound chaperone (left) and for each chaperone-containing complex (CLT and CL, right). No EC_50_ shift is observed as because TL binds with the same affinity to C as to L alone (left). Therefore, the %C_bound_ (or the %T_bound_) curve remains unchanged with an EC_50_ at 100 nM (left) despite significant CLT formation (right).

To demonstrate this, a simulation was performed under conditions of a non-cooperative system (an intrinsic cooperativity *α* = 1). A cooperativity *α* = 1 implies that the binding affinity of chaperone to ligand is the same whether the ligand is free (L) or bound to target protein (TL). Consequently, the %C_bound_ curve (or the %T_bound_ curve) would not discriminate between CL (or TL) and CLT formation, and the EC_50_ of the %C_bound_ curve would therefore remain unchanged although there is significant CLT formation. Indeed, even in conditions that favor the formation of the CLT ternary complex by increasing the concentration of available TL binary complex (200-fold excess of target protein T and a low *K*_T,1_ of 100 nM), the simulations demonstrate no shift in the %C_bound_ curve (Fig. 9). When comparing Fig. 8 and 9, it should be apparent that the shapes of the CLT formation curves are different, with Fig. 8 likely representing a more desirable profile as ternary complex formation is achieved over a broader concentration range.

**Fig. 10:**
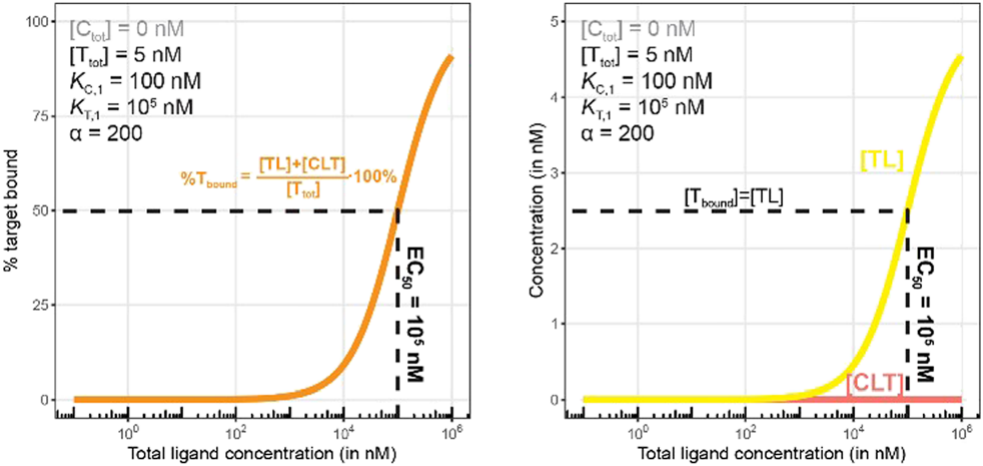
Simulations of bound target without counter protein present. Results are shown for percentage of total bound target (left) and for each target-containing complex (right). As this is a binary binding experiment, total bound target is solely due to formation of the TL complex.

**Fig. 11:**
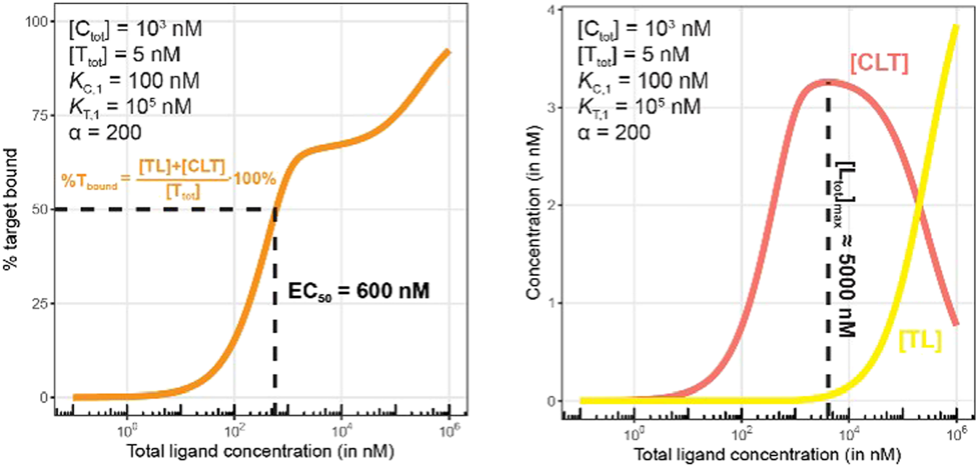
Simulations of bound target, as in Fig. 10, but now configured with an excess of the chaperone counter protein. Results are shown for percentage of total bound target (left) and for each target-containing complex (CLT and TL, right).

Fig. 10 and 11 describe a second pair of model simulations to demonstrate the importance of monitoring the apparent cooperativity through the weaker binding protein and in the presence of excess counter protein. This is intended to contrast with the first pair of simulations (Fig. 7 and 8). that monitored cooperativity through the stronger binding protein.

As in Fig. 7 and 10 is a control simulation. It shows the results of a model with no chaperone protein in the experiment ([C_tot_] = 0 nM). As this binding experiment only allows for the formation of the TL binary complex (T + L → TL), it simply serves to demonstrate how the low affinity of the target protein elicits a significantly weaker binding curve. As expected, the %T_bound_ and [TL] curves are proportional and dependent upon the binding affinity (EC_50_ = *K*_T,1_ = 100 000 nM).

The addition of the higher affinity chaperone protein at a 200-fold excess to [T_tot_] ([C_tot_] = 1000 nM) reveals a striking result (Fig. 11). The EC_50_ of the %T_bound_ curve shifts from 100 000 nM to 600 nM, which corresponds to an apparent cooperativity of 166 (100 000 nM/600 nM) compared to 2.7 when monitoring was performed with the stronger binding protein (Fig. 9). Importantly, and to highlight the robustness of this method regardless of which protein is used to monitor CLT formation, the [L_tot_]_max_ is found again at roughly 5000 nM because CLT formation is not depending on through which of the proteins (C or T) it is monitored.

Like what was observed in Fig. 8, the %T_bound_ curve (Fig. 11, left) is distinct from the CLT formation curve (Fig. 11, right). The underlying factors that govern the shape of the CLT and TL formation curves were described in Fig. 8. The observed EC_50_ of 600 nM is closer to the intrinsic *K*_C,2_, which is *K*_C,1_/*α* = 100 000 nM/200 = 500 nM, than when the EC_50_ is monitored in the same system through the stronger binding chaperone C with EC_50_ = 37 nM, *K*_T,2_/*α* = 100/200 = 0.5 nM. This shows that the assay is bottoming out earlier if the binary *K*_d_ is already low (strong binding), while the leverage effect for the EC_50_ shift is much more pronounced if the corresponding binary *K*_d_ of the monitored protein is high (weak binding).

## Summary and conclusion

In the presented work, a novel model-based method for retrieving the intrinsic cooperativity *α* from only binary dissociation constants of the ligand to its chaperone and target protein by iteratively matching one simulated and measured binding curve has been described. The proposed method is easily applicable to biochemical assays where these parameters are typically known and to cellular assays by measuring additional protein concentrations. Based on the model-based estimation of free ligand concentrations [L] from total ligand [L_tot_], the fundamental difference of concentrations of [L^well^_unbound_] inside a biochemical assay well relative to its cellular counterpart [L^cell^_unbound_] is highlighted. In addition, the intrinsic cooperativity, retrieved by the proposed workflow, together with binary dissociation constants and concentrations of ligand, chaperone and target enables the use of a mathematical model to predict ternary complex concentrations. This thereby serves to translate the biochemical potency of ternary complex-forming compounds into the corresponding fraction of cellular target protein involved into ternary complex (*i.e.*, the target occupancy).

Of note, it was demonstrated that if the apparent cooperativity is monitored through changes on the weaker binding target T, the shift in the %T_bound_ curve becomes much larger relative to the monitoring through C resulting in higher absolute numbers of apparent cooperativities, which are more accurately estimated. Furthermore, the importance of monitoring the intrinsic cooperativity *α* of ternary complex-forming compounds during their biochemical optimization to ensure higher potency, selectivity and better ADME properties has been emphasized.

Approaches to analyse molecular glues of type III that are characterized by an intrinsic affinity of the involved proteins to each other have not been discussed here. In principle, this type of glue has been studied by de Vink *et al.*^15^ but not in context of all here discussed subtypes. Our work on a mathematical model that covers all types of ternary complex-forming compounds will be discussed in due time. In addition, the described work is limited to the equilibrium situation. For application to the *in vivo* situation additional processes need to be included and could be the aim of future studies.

## Conflicts of interest

All authors were employees of Novartis at the time the work was performed.

## Supplementary Material

CB-004-D2CB00216G-s001
