# Supplementary figures and images for: A model-informed method to retrieve intrinsic from apparent cooperativity and project cellular target occupancy for ternary complex-forming compounds
Source: RSC Chem Biol. 2023 May 19;4(7):512–23. doi: 10.1039/d2cb00216g (PMC10320841; doi:10.1039/d2cb00216g)

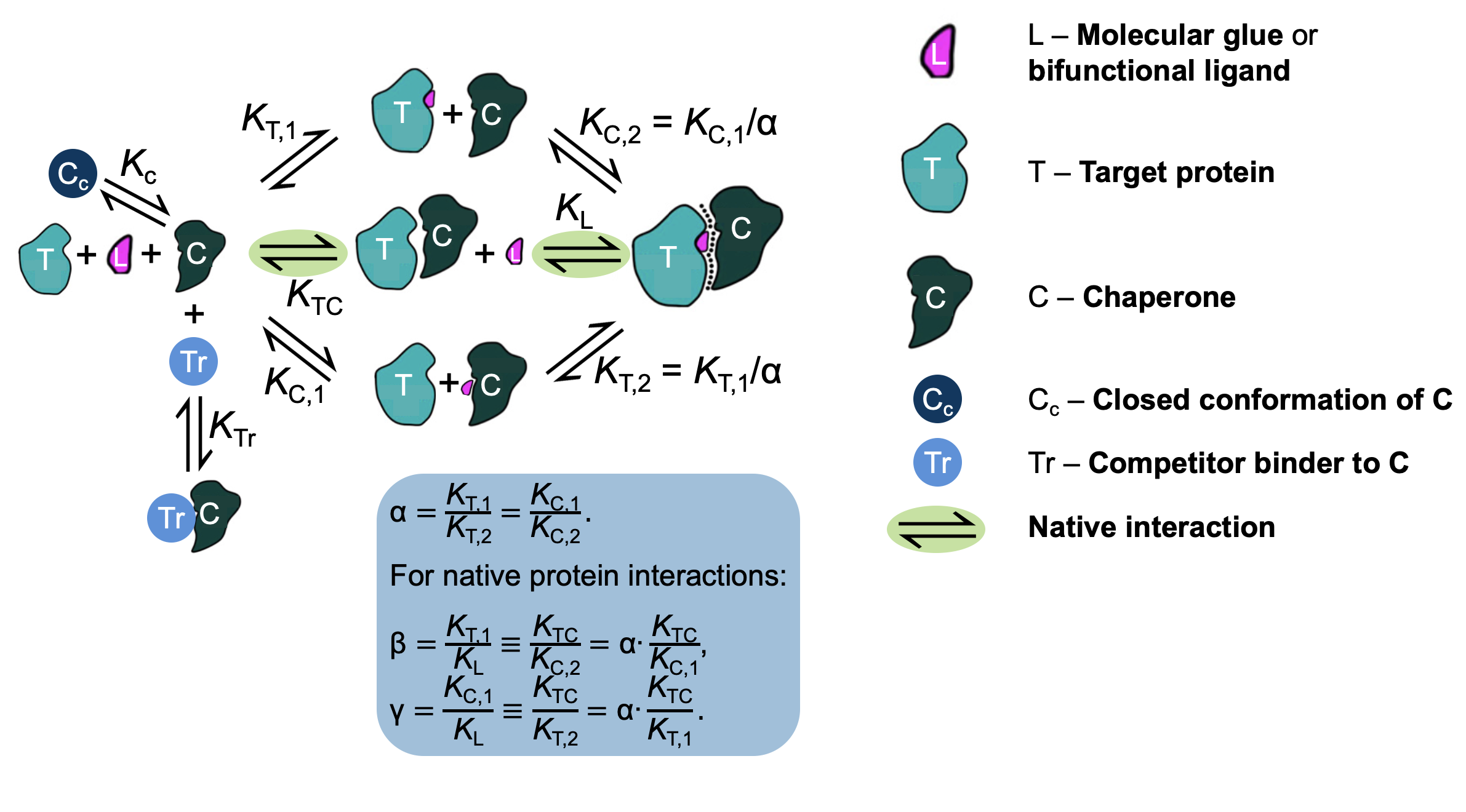

Supplement: CB-004-D2CB00216G-s001 [file CB-004-D2CB00216G-s001.zip › Ternary_Complex_Simulator_App/www/Scheme_Ternary.png]

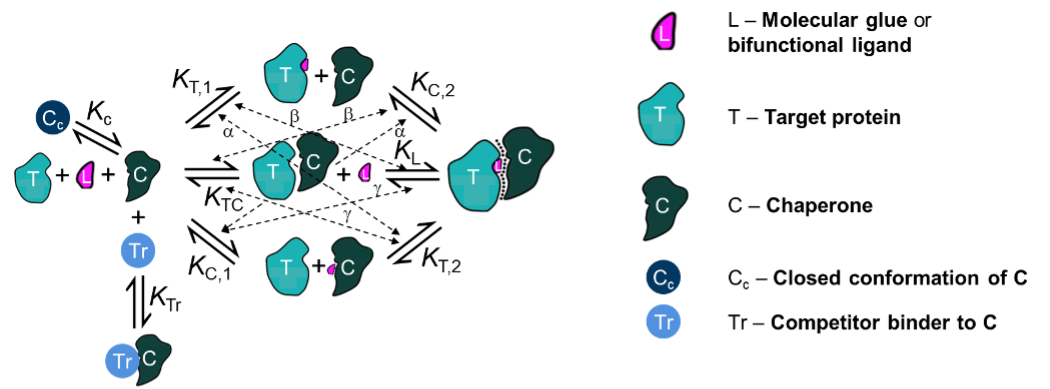

Supplement: CB-004-D2CB00216G-s001 [file CB-004-D2CB00216G-s001.zip › Ternary_Complex_Simulator_App/www/Scheme_Ternary_detailed.png]
